# Adherence to anti-malarials among patients diagnosed with malaria in East Africa: a systematic review and meta-analysis

**DOI:** 10.1186/s12936-025-05303-y

**Published:** 2025-05-03

**Authors:** Jackline D. Nkoma, Susan F. Rumisha, Hamisi S. Japhari, Emanuel L. Peter

**Affiliations:** 1 Mabibo Traditional Medicine Research Centre, National Institute for Medical Research, Dar Es Salaam, Tanzania; 2 Malaria Atlas Project, East Africa Node, Ifakara Health Institute, Dar Es Salaam, Tanzania; 3 School of Population Health, Faculty of Health Sciences, Curtin University, Bentley, WA Australia

**Keywords:** Adherence, East Africa, Malaria, Anti-malarials, Systematic review

## Abstract

**Background:**

East Africa continues to bear a significant share of the global malaria burden, despite its commitment to the malaria elimination goal of 2030. Furthermore, reported variations in adherence to anti-malarials hamper the regional effort in malaria elimination. Moreover, the region has no comprehensive and comparable adherence estimates for policymakers to set priorities, target control strategies, and evaluate the effectiveness of interventions. Hence, this systematic review synthesized the regional adherence estimate for East Africa.

**Methods:**

Authors searched articles from PubMed, Science Direct, CINHAL, Scopus, and Google Scholar. Two authors independently assessed retrieved studies for eligibility and risk of bias, then the adherence rate was pooled using the random effect model implemented in STATA. Publication bias was assessed using a funnel plot symmetry and the Egger test. Subgroup analysis was performed to explore the effect of the national and types of regimens on the overall estimate. Qualitative analysis was applied to explain factors that influence adherence.

**Results:**

A total of 29 studies with 15 927 participants were included. The overall adherence rate was 70.30% (95% CI 61.93–78.67; 29 studies; I^2^ = 99.76%), with the highest level reported in Rwanda (100%, 95% CI 97.28–100.00) and lowest in Tanzania (6.99%, 95% CI 0.2.81–11.17). Furthermore, adherence was high for chloroquine plus sulfadoxine-pyrimethamine (96.27%, 93.87–98.66; one study). Recalling correct instructions and taking the first dose at the health facility had a positive influence on patient adherence.

**Conclusion:**

On average, about three-quarters of malaria patients in East Africa adhere to their medications. In light of these findings, further interventional studies are needed to address low adherence to anti-malarials in the region. Moreover, adherence studies with the appropriate method of measurement are still needed to obtain a robust generalizable estimate in East Africa.

*Trial registration* This review was registered at PROSPERO with the registration ID CRD42023410048.

## Background

Malaria remains a global public health and developmental challenge. In 2023, the World Health Organization (WHO) indicated that about 263 million malaria cases and 597,000 deaths occurred globally [1]. Africa had a significant share of the malaria burden. In 2023, about 94% (246 million) of malaria cases and 95% (569,000) of malaria deaths were reported in the WHO African region [1]. Among them, the East African countries contributed to nearly 26% of cases. Furthermore, three of the East African countries, that is, the United Republic of Tanzania, the Democratic Republican of Congo (DRC), and Uganda remained among the 11 high burdens to high impact countries (HBHI) that accounted for 66% of global malaria cases (66%) and 68% of malaria deaths (68%) [1].

Countries in East Africa have committed to the malaria elimination goal of 2030, with many of the cases occurring in DRC and Uganda [2, 3]. Despite the commitment and availability of national malarial strategic plans [4–6], countries in the region differ in the speed toward the elimination goal due to the dynamicity of social-cultural, biological, environmental, and health system factors [2].

Currently, countries in the region have stepped up investment aimed at increasing the number of health facilities, early malaria diagnosis, supply of insecticide-treated bed nets, timely treatment with anti-malarial, including artemisinin-based combination therapy (ACT), East Africa cross-border collaboration against malaria interventions, among other strategies.

However, studies indicated that, due to the bulk of tablets, patients prescribed ACT are less likely to adhere to their medications [7]. This non-adherence to the recommended treatment schedule might lead to malaria recurrence, treatment failure that prevents countries from reaching their elimination targets, and even the occurrence of drug resistance [8]. Furthermore, non-adherence affects households and countries' economies, increases the burden on healthcare systems, and contributes to absenteeism which affects both school and work performance [9, 10].

Studies pointed out that adherence to medications is influenced by several factors, including, geographical location, patient’s age, assessment methods, and type of drug regimen. For instance, a study conducted in Kampala, Uganda showed an adherence to anti-malarial of 65.8% [11] while that of South Kivu, DRC showed an adherence of 75% [12]. Similarly, another study conducted in a rural community in Tanzania showed an adherence rate of 72% among health facility patients [13]. Although, several isolated individual studies reported adherence to anti-malarials among the East Africa countries, a regional comprehensive and comparable adherence estimate is lacking. Hence, a need to systematically synthesize regional adherence to establish a comprehensive and comparable estimate. Information obtained could offer an opportunity for national malaria programmes and regional cross-border malaria programmes to direct tailored intervention efforts in poor adherence countries that continue to pose challenges in national and regional malaria elimination.

## Methods

### Registration

The authors prepared a systematic review and meta-analysis Protocol according to the Preferred Reporting Item for Systematic Review and Meta-analysis (PRISMA-P) guideline [14]. Then, the study protocol was registered in PROSPERO with the registration number CRD42023410048. Finally, the authors have reported review findings according to the Preferred Reporting Item for Systematic Review and Meta-analysis (PRISMA) and the PRISMA abstract checklist [15].

### Search strategy and selection criteria

Authors searched PubMed, Science Direct, CINHAL, Scopus, and Google Scholar from inception to 30 December 2023. The reference lists of the retrieved studies were screened for additional studies not captured in a primary search. The search was re-run just before the final analysis to capture the most recent published articles. The search strategy involved a combination of MeSH terms and keywords that were divided into three components; population, intervention, and output. The population component had the following terms; “Kenya”, “Uganda”, “Tanzania”, “United Republic of Tanzania”, “Rwanda”, “Burundi”, “Democratic Republic of Congo”, “DRC”, “South Sudan”, and “east Africa”. The intervention had “Anti-malarial*”, “Anti-malarial”, “malaria medicine”, “medication”, “malaria drug”, and “therapy” while output had “adherence”, “patient adherence”, “patient compliance”, “medication adherence” and “compliance”. The search components were combined using appropriate Boolean operators. Throughout the search, language was not restricted. The exclusion criteria were; (i) qualitative studies without numerical estimate of adherence, (ii) all types of reviews, and (iii) studies without full text. After the application of the search strategy, all studies were imported into the Mendeley reference manager for duplicate removal and screening. Two authors JDN and HSJ independently screened the title and abstract using pre-determined inclusion and exclusion criteria. The ELP resolved the disagreement between the first two authors. Then, JDN and HSJ independently screened the full text of selected studies. Successful articles were included in the final data extraction. The authors used the Joanna Briggs Institute critical appraisal tools (JBI) for risk of bias assessment.

### Data extraction and analysis

A standardized data extraction tool was created in Epi-data Manager. Five studies were used to test the tool, then adopted after necessary changes were made. Data extracted included; study design, location, sample size, drug regimen, method used to assess adherence, approach used to assess adherence, adherence rate, adherence rate without intervention, adherence rate with intervention, intervention, definition of adherence, and age. Quantitative synthesis was performed using Stata version 17. The I^2^ index was used to evaluate the heterogeneity severity of included studies. A random effect model was used to combine data with a 95% confidence interval. Publication bias for each outcome was assessed by testing the asymmetry of the funnel plot using Egger’s test [16]. For the publication bias assessment, a meta-analysis of ten or more studies was considered because test power is generally too low to distinguish chance from real asymmetry when it includes a smaller number of the primary studies [16, 17]. When publication bias was detected, the trim and fill method was used to correct the probable publication bias by imputing missed studies and adjusting the effect size [18]. Qualitative synthesis was performed by systematically organizing the information extracted from the included studies.

## Results

### Study selection flow

**Fig. 1:**
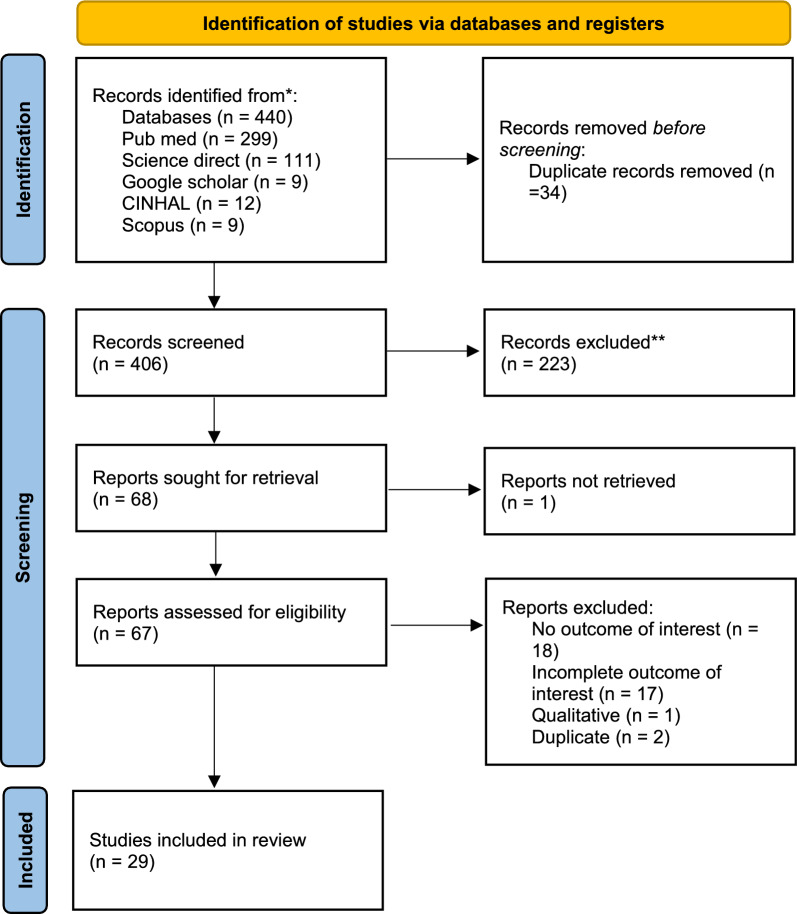
PRISMA chart showing study selection

In total, about 276 studies were retrieved (Fig. 1). When duplicates were removed 267 studies remained for title and abstract screening. About 223 studies were excluded during the title and abstract screening. From the 44 studies remaining, the full text of only 43 studies were retrieved and assessed, while 20 studies were excluded. Finally, 23 studies were included in the review.

### Study characteristics

Of the 29 studies included, Uganda and Tanzania have 10 and nine studies respectively, Kenya has seven studies, while DRC, South Sudan, and Rwanda have one study each. The use of artemisinin-based combinations was high by 87.1% (27 studies), compared to non-artemisinin-based combinations. Furthermore, the majority of the studies 14 (48.28%) used multiple methods to assess adherence, while the remaining use the remaining used a single method of assessment, including, pill count 4 (13.79%) and self-report 11 (37.93%) (Table 1).

**Table 1:** Characteristics of studies included in the antimalarials usage adherence

Author, Pub year	Country	Study design	Treatment regimen	Sample size	Adherence rate (%)	Method	Approach
Achan, [40]	Uganda	Randomized trial	AL & Quinine	85 & 75	95 & 85	Self-report & pill count	Completed treatment and timely completion
Ali, [41]	Tanzania	Cohort	Dihydroartemisinic/ piperaquine phosphate, artemisinin, piperaquine, and artesunate amodiaquine winthrop	2009	90	Self-report	Complete treatment
Amin, [42]	Kenya	Randomized trial	SP & AQ	172	SP 66.7%, AQ 13.8%	household survey questionnaire	High dose/low dose consumption
Baiden, [43]	Tanzania	Cohort	Dihydroartemisinin/piperaquine	1100	95	Self-report	Complete treatment
Bawate, 2024 [44]	Uganda	Longitudinal	ACT	844	69.7	self-report & pill count	complete treatment
Beer, [45]	Tanzania	Cross-sectional	AS/AQ	174	77	Self-report & pill count	Complete treatment
Bruxvoort, [13]	Tanzania	Descriptive(facility) and cluster-randomized trial	AL	1022	72	Pill count	Completed treatment and timely completion
Bruxvoort, [27]	Tanzania	Randomized trial	AL	451	70	Self-report and pill count	Complete treatment
Bruxvoort, [46]	Tanzania	Randomized trial	AL	696	67	Self-report & pill count	Complete treatment
Cohen, [20]	Uganda	Experimental	ACT	1702	64	Self-report	Complete treatment
Cohen, [47]	Uganda	Randomized trial	AL	152	66	Self-report & pill count	Complete treatment
Fogg, [48]	Uganda	n/a	AL	210	90.0	Self-report, pill count, lumefantrine assay3	verified timely complete treatment
Gerstl, [12]	Democratic Republic of Congo	Experimental	AS/AQ	148	75	Self-report & pill count	Complete treatment & verified complete treatment
Gore-Langton, [21]	Kenya	Cohort	AL	195	60	Self-report & pill count	Complete treatment & verified complete treatment
Idris, [8]	South Sudan	Case–control	ACT	289	26	Self-report	Complete treatment
Kachur, [49]	Tanzania	Randomized trial	SP plus AS	128	75.0	Self-report & pill count	complete treatment
Kalyango, [50]	Uganda	Randomized trial	AL	667	96	Self-report & pill count	Complete treatment
Kolaczinski, [51]	Uganda	Observation study	chloroquine plus SP	241	96	Self-report	Complete treatment
Lawford, [52]	Kenya	Observation study	AL	918	64	Pill count	Complete treatment
Minzi, [19]	Tanzania	Descriptive study	AL	143	7	Self-report & pill count	Timely completion
Morris, [53]	Tanzania	Randomized trial	Dihydroartemisinin -piperaquine (DP) and single low dose (SLD) of primaquine	1136	82	Self-report	Complete treatment
Mutua, [54]	Kenya	cross sectional	AL	492	93.7	Self-report	complete treatment
Nshakira, [55]	Uganda	n/a	chloroquine	426	37.8	self-report	complete treatment
Onyango, [56]	Kenya	Cross sectional	AL	297	47	Self-report	Timely completion
Saran [11]	Uganda	Randomized trial	AL	1018	66	Pill count	Verified complete treatment
Talisuna, [57]	Kenya	Randomized trial	AL	562	71	Self-report & pill count	Complete treatment
Twagirumukiza, [58]	Rwanda	Randomized trial	Quinine	54	100	Self-report, manual pill count and electronic pill count	Timely completion
Wasunna, [31]	Kenya	Cross sectional	AL	147	69	Self-report	Complete treatment
Yeka, [59]	Uganda	Randomized trial	AS/AQ & AL	374	100	Pill count	Complete treatment

*AL* Artemether Lumefantrine, *SP* sulfadoxine-Pyrimethamine, *AQ* Amodiaquine, *AS* artesunate

### Definition of adherence and measurement methods

Adherence definition varies between studies, the majority (n = 20) of the studies have defined adherence as completing the prescribed dose. Other eight studies defined it as taking the correct dose at the correct time. While only one study has defined adherence by the percentage of the amount of medicines consumed. In this review, adherence was defined as taking a dose as prescribed by a drug dispenser. A patient was addressed as adherent when blister packs were found empty on the day of the interview, and/or has reported he/she has completed a dose.

### Adherence to anti-malarial

**Fig. 2:**
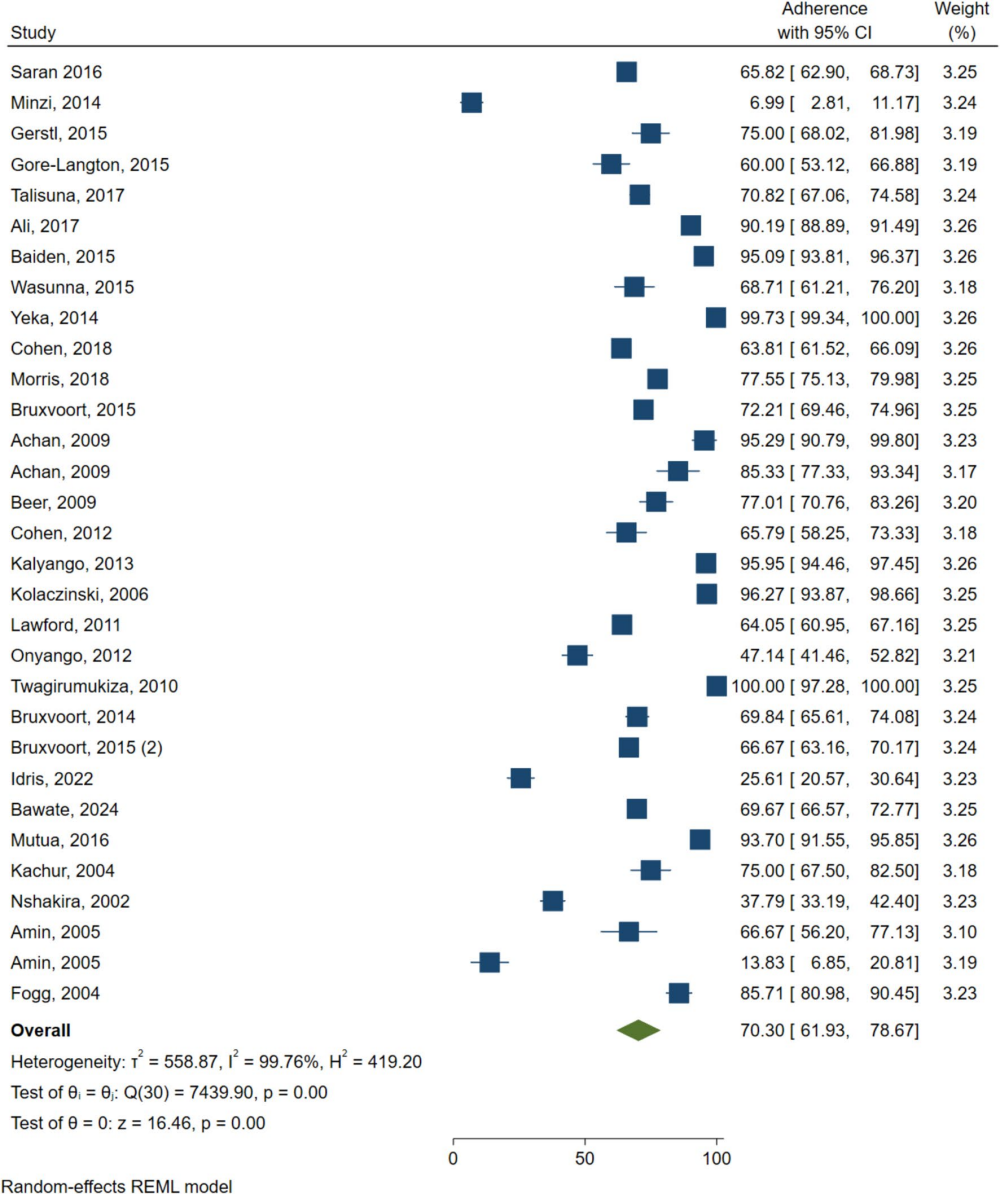
Forest Plot for overall antimalarial adherence in East Africa

The overall average adherence rate was 70.30% (95% CI 61.93–78.67; 29 studies; I^2^ = 99.76%) Fig. 2. It was observed that Rwanda reported the highest adherence rate at 100% (95% CI 97.28–100.00). Followed by a study conducted in Uganda, which reported an adherence rate of 99.73% (95% CI 99.34–100.00). while Tanzania had the lowest adherence rate at 6.99% (95% CI 2.81–11.17).

### Sub-group analysis

**Fig. 3:**
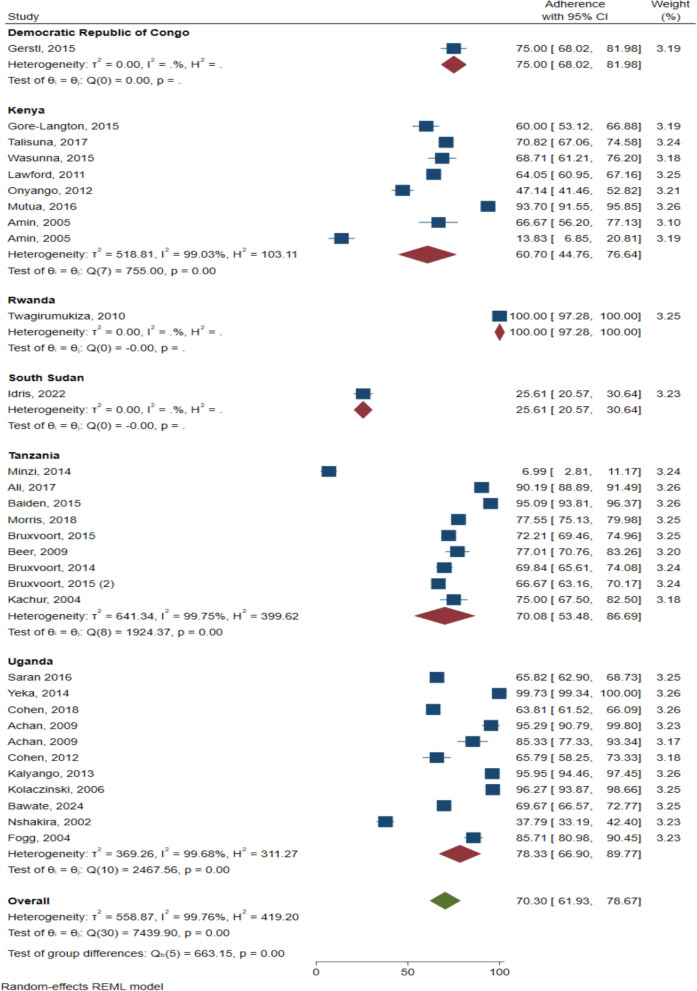
Anti-malarial adherence by country

**Fig. 4:**
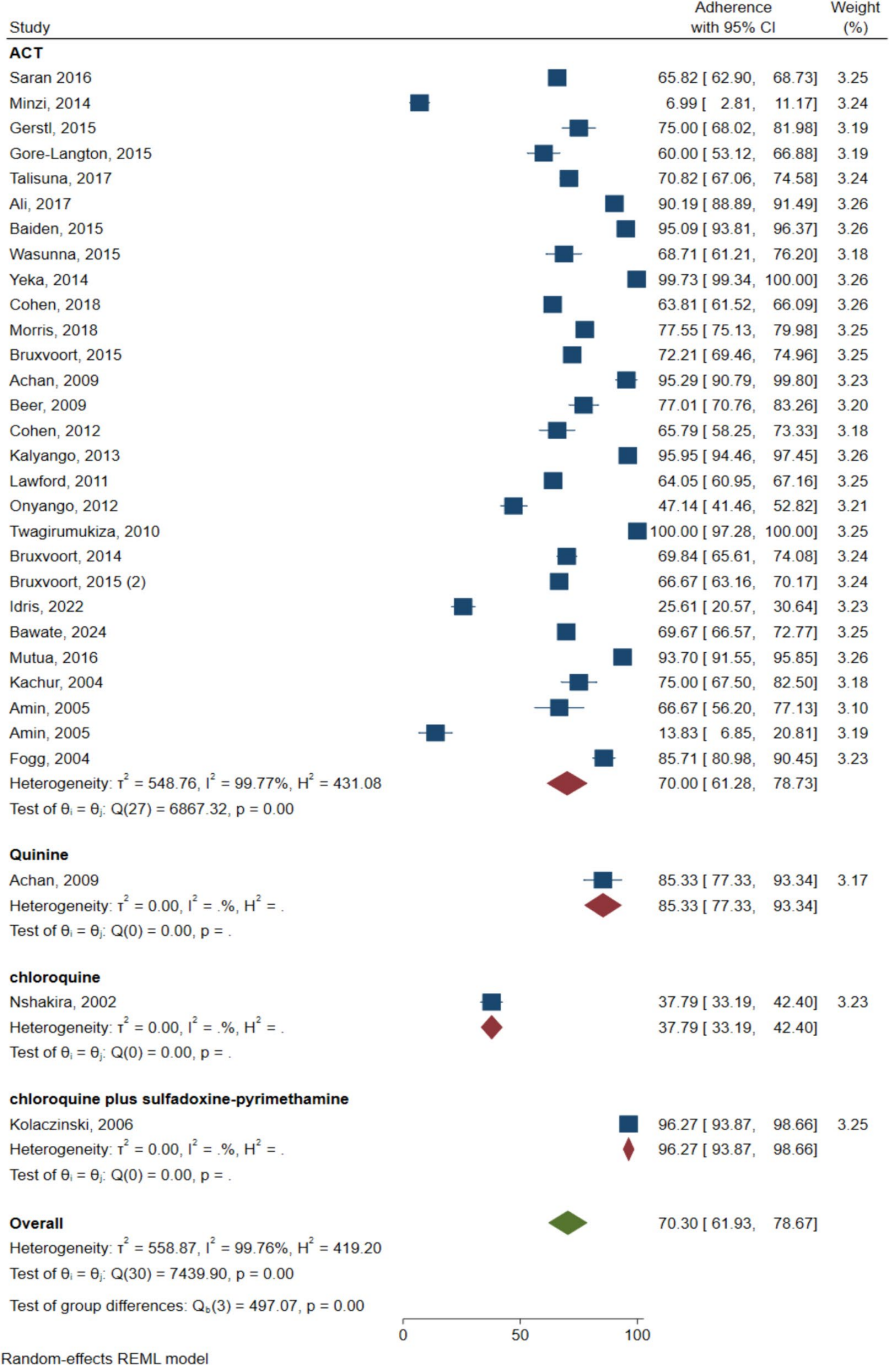
Anti-malarial adherence by treatment regimen

The sub-group analyses were performed by country (Fig. 3), and by treatment regimen (Fig. 4). At the national level, the highest anti-malarial adherence rate was reported in Rwanda 100% (95% CI 97.28–100.00; one study) Fig. 3. Followed by Uganda (78.33%, 95% CI 66.90–89.77; 10 studies, I^2^ = 99.68), while the lowest anti-malarial adherence was reported in South Sudan (25.61%, 95% CI 20.57–30.64; one study) (Fig. 3).

High anti-malarial adherence was observed among those on chloroquine plus sulfadoxine-pyrimethamine (96.27%, 95% CI 93.87–98.66; one study). While it is low with chloroquine (37.79%, 95% CI 33.19–42.40; one study) (Fig. 4). Adherence rate among the patients who use ACT was (70%, 95% CI 61.28–78.73; 27 study, I^2^ = 99.77).

### Publication bias and sensitivity analysis

**Fig. 5:**
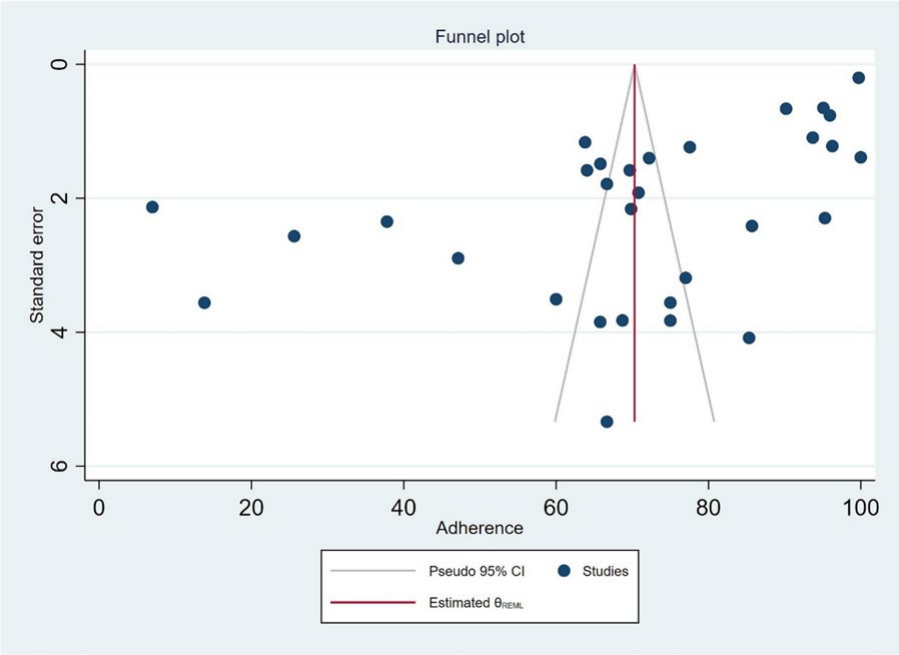
Publication bias of the studies included

The funnel plot showed a significant substantial asymmetry (Fig. 5). The Egger’s test was done to confirm the presence of publication bias. Since the Egger’s (p = 0.0325) showed the existence of small study effect, while trim and fill method does not change results significantly, then small-study effects may be due to true heterogeneity rather than publication bias. Sensitivity analysis was performed with the random effect model to see the effect of a single study on the overall estimate.

**Fig. 6:**
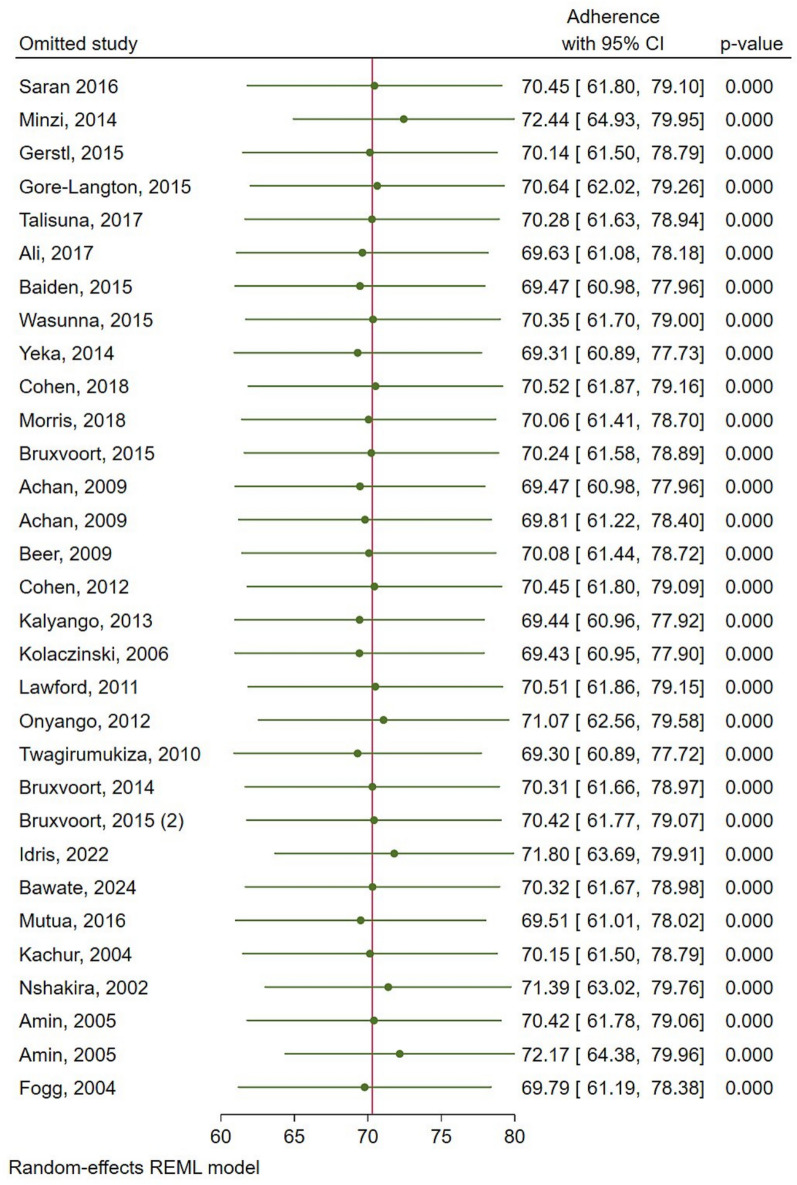
Sensitivity analysis for anti-malarial adherence studies in East Africa

The omission of Minzi, 2014, Idriss, 2022 and Amin, 2004 (with amodiaquine) seems to have a relatively larger influence compared with other studies, an increase of roughly 0.02 on the overall anti-malarial estimate (Fig. 6).

### Qualitative assessment

Among the 23 included studies, only 11 have reported factors associated with adherence. Within the 11 studies included, a total of 13 factors were found to influence patient adherence (Table 2). This analysis found that patients aged of less than 13 years were found to adhere as opposed to those who are more than 13. Patients with high education level, ability to read and high income, and female were significantly associated with high level of adherence.

**Table 2:** Factors associated with adherence

Factors	Narratives	citation
Demographic		
Age	Respondents aged less than 13 years were more likely to adhere compared to those who were more than 13 years old. As age increases, the adherence rate decreases	[19, 45, 47, 56]
Sex	Females adhere more compared to males	[19, 47, 56]
Education	Patients who could read, and attained secondary or beyond education were more likely to complete treatment than others	[19, 45, 47, 56]
Income	Patients from high-income status were more likely to complete treatment than those from lower income	[56]
Patient and medication-related factor		
Taking the first dose at the outlet	Patients who take their first dose at a public health facility were more likely to finish their medications on time as prescribed by the dispenser	[19, 45, 46]
Patients who heard ACTs at baseline	Patients who heard ACTs at the study baseline were more likely to adhere compared to those who didn’t hear it	[20]
Improved conditions	Patients who felt well are less likely to adhere to their medications compared to those who still feel unwell on the second day	[11, 47, 50]
Long time between obtaining AL and an interview	The time between obtaining AL and the interview was associated with completing treatment	[13]
RDT positive results	MRDT positive results didn’t significantly increase adherence	[11]
Short message	The use of sticker messages contributes to an increase in adherence rate	[20, 27]
Use of packaging to explain how to take AL	The use of packaging to explain how to take their medication was associated with timely completion	[13]
Understanding given instructions	Recalling the correct instruction given by the dispenser was associated with adherence	[13, 21, 50]
Seeking care behaviour and care givers perceptions	Seeking care within two days of fever was associated with timely completion	[13, 50]

Two studies assessed drug intake behaviour [13, 19]. It was found that taking the first dose at a public health facility improves adherence. The use of short messages (sticker messages) is associated with a high level of adherence [20]. Recalling the number of correct instructions on how to take AL was also found significantly associated with adherence [21]. There is weak evidence that positive malaria results lead to an increase in the total number of pills a patient has consumed [11]. The use of packaging to explain how to use AL is associated with timely completion.

## Discussion

On average, adherence to anti-malarials in the East Africa region was 72% with great variations observed ranging from seven percent to 100%. Although there is no standard cut-off point for optimal adherence for anti-malarial, several studies pointed out that adherence levels of 80% and above are associated with desirable clinical outcomes for different therapeutic types [22, 23]. The observed sub-optimal adherence to anti-malarials in this review implies that regional effort to eliminate malaria is at stake and calls for national malaria programmes to direct tailored intervention in poor adherent countries to achieve the elimination goal of 2030 [4, 6].

The observed high heterogeneity in these estimates highlights the inherent differences in the included studies. Differences in study design, the definition of adherence, methods of assessment, geographical differences and study population could have influenced adherence rates reported. Additionally, the changes in healthcare systems, cultural behaviour, level of education, disease burden, and social economics factors could also have some influence on the observed heterogeneity. Since almost all of the studies used self-reported method, results have to be used with caution. A self-report method may result in unrealistic estimates of adherence level due to recall bias, social desirability and failure to indicate the actual time that medicine was taken [24–26].

The observed wide variation of adherence to anti-malarials reported in our study is similar to those documented in previous systematic review of adherence to anti-malarials globally [27]. Several factors contribute to anti-malarial adherence variations as reported in the previous studies, including, method of assessment, definition of adherence, pill load, influence of herbal medicine, and patient social economic status are some of them [7, 28–31]. These factors could have contributed to the observed variation reported in this review.

The majority of the included studies had adherence rate greater than 50% while only one had a small adherence of 7% in Tanzania, with the highest in Rwanda having 100%. Despite the similarity in methods of assessing adherence, still there is a wide variation between the highest and the lowest observed adherence levels. The differences in age and education level of participants between the two studies might influence their wide variation. A study conducted by Minzi in Tanzania included participants with at most primary education regardless of their age. While a study conducted by Twagirumukiza in Rwanda included participants aged less than 5 years. Moreover, a study conducted by Minzi was in rural areas, Twagirumukiza’s study was conducted in urban (Rwanda University Hospital of Butare). Malaria prevalence might also influence the variation between the two studies. Whereby, Tanzania is among the 11 countries with the highest malaria burden. While Rwanda is among the countries with minimal malaria cases and deaths.

However, this observation included only six out of seven East African countries that contributed the data. Furthermore, Uganda, Tanzania, and Kenya had more articles than those obtained from Rwanda, South Sudan, and the Democratic Republic of Congo which contributed only one each. The difference in the amount of data included could impede adequate comparison of the level of adherence among regional countries. Nevertheless, these variations in adherence by countries could be used as proxy indicators to inform the likelihood of achieving the malaria elimination goal of 2030. Essentially, countries with low levels of anti-malarial adherence could delay reaching the malaria elimination goal. In Uganda, multiple medicines were used from eight different studies, but both ACT and non-ACT had reported an adherence rate of more than 50%. This means type of medicine may not affect adherence [32].

Moreover, the use of short messages is associated with a high level of adherence [20]. The use of SMS remainder was found to be effective in the study conducted in Uganda. The use of SMS reminders was also found effective in different studies that aimed to boost the adherence level of medications and guidelines [33–37]. A study conducted at Kenya proved the effectiveness of SMS remainder in improving antiretroviral therapy (ART) adherence among patients [37]. The use of SMS to remind patients to take their medicine was also effective in Kenya [36]. These findings highlight the potential for scalability, given the widespread availability of mobile phones in East Africa. However, challenges such as illiteracy, language diversity, and ensuring privacy must be addressed for broader implementation.

Lastly, the use of drug packaging to explain how to use medicine was effective in busting medication adherence. A similar method was used in Uganda where patients raised their adherence rate and reduced the number of non-adherence [38]. In Rwanda, the improvement of the anti-retroviral drug package helped patients improve their treatment schedule more effectively [39].

## Conclusion

On average, about three-quarters of malaria patients in East Africa adhere to their medications. In light of these findings, the authors recommend further interventional studies to address poor adherence to anti-malarial in the region. Moreover, adherence studies with appropriate assessment methods are still needed, particularly among countries that had limited adherence data to obtain a robust generalizable estimate in East Africa.

## Data Availability

All data used to support the findings and materials will be available on request.

## Abbreviations

JBI: Joanna Briggs Institute
ACT: Artemisinin-based combination therapy
MOH: Ministry of Health
WHO: World Health Organization
FDA: Food and Drug Administration
PRISMA: Preferred Reporting Item for Systematic Review and Meta-analysis
CI: Confidence interval
RDT: Rapid diagnostic test
ADDO: Accredited Drug Dispensing Outlets
AL: Artemether Lumefantrine
SP: Sulfadoxine-pyrimethamine
AQ: Amodiaquine
AS: Artesunate
